# 

**Published:** 1896

**Authors:** 


### Treatment of Typhoid Fevers,

WITH A FEW ADDITIONS.

A PART OF THE

### ANALYTICAL THERAPEUTICS

BY

CONSTANTINE HERING, M. D.

SECOND EDITION.

Philadelphia, Pa.:
THE HOMŒOPATHIC PHYSICIAN,
1231 Locust Street.
1896.

Copyright, 1896, by Walter M. James, M. D.

